# Traditional knowledge of wild edible plants used in the northwest of the Iberian Peninsula (Spain and Portugal): a comparative study

**DOI:** 10.1186/1746-4269-3-27

**Published:** 2007-06-07

**Authors:** Manuel Pardo-de-Santayana, Javier Tardío, Emilio Blanco, Ana Maria Carvalho, Juan José Lastra, Elia San Miguel, Ramón Morales

**Affiliations:** 1 Departamento de Biología (Botánica), Universidad Autónoma de Madrid, c/Darwin 2, Campus de Cantoblanco, E-28049 Madrid, Spain; 2 Real Jardín Botánico de Madrid, Plaza de Murillo, 2. E-28014 Madrid, Spain; 3 Instituto Madrileño de Investigación y Desarrollo Rural, Agrario y Alimentario, Apdo. 127, 28800 Alcalá de Henares, Madrid, Spain; 4 Escola Superior Agraria, Campus de Sta. Apolónia, 5301-855 Bragança, Portugal; 5 Universidad de Oviedo. C/Catedrático Rodrigo Uría s/n E-33071 Oviedo, Spain

## Abstract

**Background:**

We compare traditional knowledge and use of wild edible plants in six rural regions of the northwest of the Iberian Peninsula as follows: Campoo, Picos de Europa, Piloña, Sanabria and Caurel in Spain and Parque Natural de Montesinho in Portugal.

**Methods:**

Data on the use of 97 species were collected through informed consent semi-structured interviews with local informants. A semi-quantitative approach was used to document the relative importance of each species and to indicate differences in selection criteria for consuming wild food species in the regions studied.

**Results and discussion:**

The most significant species include many wild berries and nuts (e.g. *Castanea sativa, Rubus ulmifolius, Fragaria vesca*) and the most popular species in each food-category (e.g. fruits or herbs used to prepare liqueurs such as *Prunus spinosa*, vegetables such as *Rumex acetosa*, condiments such as *Origanum vulgare*, or plants used to prepare herbal teas such as *Chamaemelum nobile*). The most important species in the study area as a whole are consumed at five or all six of the survey sites.

**Conclusion:**

Social, economic and cultural factors, such as poor communications, fads and direct contact with nature in everyday life should be taken into account in determining why some wild foods and traditional vegetables have been consumed, but others not. They may be even more important than biological factors such as richness and abundance of wild edible flora. Although most are no longer consumed, demand is growing for those regarded as local specialties that reflect regional identity.

## Background

There has been renewed or increasing interest in consuming wild food plants [e.g. [1-7]]. Despite agricultural societies' primary reliance on crop plants, the tradition of eating wild plants has not completely disappeared, their nutritional role and health benefits being reported in many surveys worldwide [e.g. [8-18]].

In Europe, they were important as dietary supplements, providing trace elements, vitamins and minerals. Nowadays, however, consumption is determined less by calory input and more by the pleasure of gathering wild resources, recreating traditional practices and enjoying characteristic flavours [19-28].

Most studies of wild edible plants focus on function within one culture or ethnic group, there being only few papers that compare food plants of various cultures [29-31]. However, some papers have compared medicinal floras and other useful plants [32-36]. Such comparative studies contribute to understanding why edible species are consumed or rejected and can provide interesting insights into food selection criteria.

In this paper, the term 'wild' refers to non-cultivated plants gathered in the field [see [37]]. Although most species belonging to wild food plant taxa are native, some introduced species have become feral. Certain consumed species derive from both wild and cultivated specimens, but in such cases all use-reports were considered regardless of the origin of the specimens. For example, although native only to some of our survey areas, *Laurus nobilis*, *Corylus avellana*, *Tilia platyphyllos*, *Rubus idaeus*, *Prunus avium *and *Castanea sativa *are cultivated and used at the other sites.

Given the dramatic loss of traditional knowledge regarding wild edible plants and the fact that many of the plants cited in this paper are no longer consumed, our aim is to evaluate the knowledge, diversity and cultural significance of wild edible plants used in six rural areas of the northwest of the Iberian Peninsula, comparing the cultural importance of edible taxa historically gathered as food, thereby enhancing the value of such locally produced food sources.

## Methods

### Study sites

**Figure 1:**
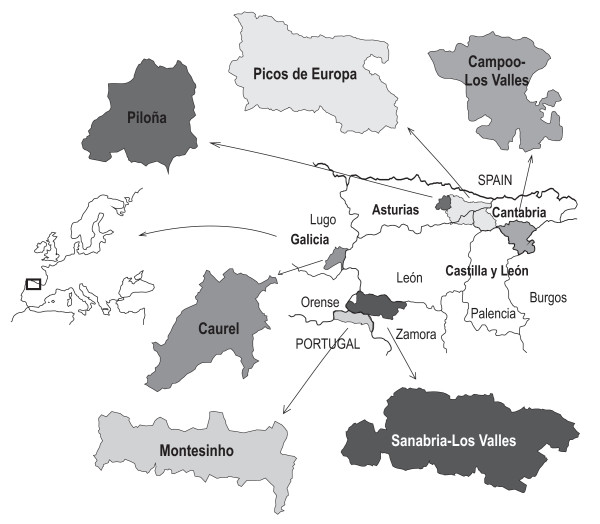
Localization of the six survey sites in the northwest of the Iberian Peninsula.

All research mentioned in this paper was conducted in six rural and mountainous areas of the northwest of the Iberian Peninsula. In Spain: *Campoo *in the south of the Cantabria Autonomous Region [38]; *Picos de Europa*, a geographical region that straddles the autonomous administrative regions of Asturias, Cantabria and León province in the Autonomous Region of Castilla y León [[39], authors personal observations]; *Piloña *in central-eastern Asturias [40]; *Caurel*, in the south-east of Lugo province (Autonomous Region of Galicia) [41]; and *Sanabria *in the north-west of Zamora province (Autonomous Region of Castilla y León) [42]. In north-eastern Portugal: *Montesinho *[43] adjoining Zamora province (Figure 1).

All six survey sites are culturally and biologically rich and most lie in protected areas, e.g. Picos de Europa National Park, Sanabria Lake Natural Park and Montesinho Natural Park. Bordering both Mediterranean and Eurosiberian floristic regions, the six sites have climates that vary from oceanic (wet and relatively mild) in Picos de Europa, Piloña, Caurel and the north of Campoo to wet-Mediterranean (drier in summer) in Sanabria, Montesinho and most of Campoo.

Landscapes include a mosaic of meadows, forests, rivers and high mountain vegetation growing on varied geological materials and soils. The predominant vegetation consists of beech (*Fagus sylvatica *L.) forest, several oak species, e.g. *Quercus robur *L.*, Quercus petraea *(Matt.) Liebl., *Quercus pyrenaica *Willd.,*Quercus faginea *Lam. and *Quercus ilex *L., chestnut (*Castanea sativa *Mill.), broom scrubland consisting of *Cytisus scoparius *(L.) Link, *Cytisus multiflorus *(L'Hér.) Sweet, *Genista florida *L., and heath comprising *Erica cinerea *L.*, Erica vagans *L.*, Erica australis *L.*, Erica umbellata *Loefl. ex L.*, Calluna vulgaris *(L.) Hull. *Fagus sylvatica*, *Quercus robur *and *Quercus petraea *are more common in the wetter northern areas, while *Quercus pyrenaica, Quercus faginea *and *Quercus ilex *grow in drier areas.

Until a few decades ago the survey sites' economies were based on agriculture, cattle rearing and several less important activities. Most of the population engaged in traditional stock farming involving few animals. Short-distance vertical transhumance and long-distance southwards transhumance of cattle and sheep were particularly important in the Cantabrian Mountains. In regions such as Campoo, low salaries meant that even people working in the steel, cement and glass industries combined wage labour with livestock farming. The largely subsistence-based household economy was boosted with income from the sale of animals, eggs, butter and handicrafts. Other important economic activities were smuggling and forestry in Montesinho, and door-to-door hawking in Sanabria, chiefly using mules.

Many fields once used to grow cereals (for bread), pulses, turnip and potatoes now provide grazing for cattle. Agriculture plays only a minor role and new economic activities, such as rural tourism, are increasingly important.

### Ethnobotanical data collection and analysis

Ethnobotanical information was obtained through informed consent semi-structured interviews with key informants over the last twenty years (1989–2004) (Table 1). Informants with a sound traditional knowledge of useful wild plants, mostly elderly long-time residents, were interviewed. Open questions about wild food consumption sought to ascertain knowledge about past and present-use, mode of consumption and preparation, collection time and collection sites for each species [44].

**Table 1:** Number of informants, localities visited and geographical features of each area

Survey sites	Informants	Localities	Surface (Km2)	Population
Campoo	107	42	1012	23000
Caurel	39	19	100	1200
Picos de Europa	131	67	1920	19900
Piloña	94	51	283	8600
Montesinho	90	30	734	7427
Sanabria	44	20	2120	15000

For this study, data were grouped into the following categories of edible plants based on folk perceptions: "vegetables", plants whose leaves, stems or even unripe fruits or seeds were consumed; "wild fruits", plants whose fruits or seeds were consumed when ripe; home-made "liqueurs" or other alcoholic drinks; "herbal teas", used in general as a *digestif*; plants used for "seasoning"; and finally, "flowers" and "underground organs", eaten for their sweetness.

Every plant species mentioned by an informant within one use-category was counted as one use-report (UR) [see [45]]. For instance, the raw fruits of *Prunus spinosa *in Picos de Europa were reported as consumed by 17 informants and used in liqueurs by 21, totalling 38 UR. However, a total number of 27 informants cited the species as useful since some informants reported use both for liqueurs and for raw consumption of fruits. We have rejected species with only one UR because such data are less reliable and sometimes dubious for drawing comparisons.

Voucher specimens were deposited at the herbaria of the Royal Botanical Garden of Madrid (MA, Real Jardín Botánico), the University of Oviedo (FCO, Universidad de Oviedo) and the School of Agricultural Engineering at Bragança (BRESA, Escola Superior Agrária).

To estimate the cultural significance of each species, we used the Cultural Importance Index (CI), whose definition and use are discussed in another paper [see [46]], with the following formula:

CI=∑i=1i=NUURiN
 MathType@MTEF@5@5@+=feaafiart1ev1aaatCvAUfKttLearuWrP9MDH5MBPbIqV92AaeXatLxBI9gBaebbnrfifHhDYfgasaacH8akY=wiFfYdH8Gipec8Eeeu0xXdbba9frFj0=OqFfea0dXdd9vqai=hGuQ8kuc9pgc9s8qqaq=dirpe0xb9q8qiLsFr0=vr0=vr0dc8meaabaqaciaacaGaaeqabaqabeGadaaakeaacqWGdbWqcqWGjbqscqGH9aqpdaaeWbqaamaalaaabaGaemyvauLaemOuai1aaSbaaSqaaiabdMgaPbqabaaakeaacqWGobGtaaaaleaacqWGPbqAcqGH9aqpcqaIXaqmaeaacqWGPbqAcqGH9aqpcqWGobGtcqWGvbqva0GaeyyeIuoaaaa@3F4E@

The index, which is based on previous indices [47,48] was obtained by adding the *UR *in every use-category (*i*, varying from only one use to the total number of uses, *NU*) mentioned for a species, divided by the number of informants in the survey (*N*).

The CI was calculated for each region. For example, *Foeniculum vulgare *in Montesinho was reported as used in liqueurs by 10 informants, for seasoning by 32 and for herbal teas by 23. The total number of survey participants was 90.

CI_*Foeniculumvulgare *_= 10/90+32/90+23/90 = 0.11+0.36+0.26 = 0.722

This additive index takes into account the spread of use (number of informants) for each species and versatility, i.e. diversity of edible uses. The theoretical maximum value of the index is the total number of different edible use categories.

A mean Cultural Importance Index (mCI) of the species was used to assess wild food plant use in the Peninsular northwest as a whole. It is also useful in evaluating CI differences among the various sites. Since a null value may be due to either the species not growing in the area or growing but not being consumed, the mean value preferably needs to be calculated by considering only regions where the species grows and is available. For example, if the null values of the areas where it does not grow (Sanabria and Montesinho) are rejected, the mean value for *Fagus sylvatica *is 0.055; however, the figure decreases to 0.037 if all six areas are considered. Thus, the mean value takes into account species selection or rejection and availability.

To measure the cultural importance of families (CIf), we added the CI of the species from each family, following Galeano [49]. We preferred using the sum instead of the mean as proposed by Phillips and Gentry [48] so as to highlight more diverse families which would otherwise be underestimated.

When comparing the edible floras of different regions, it is crucial to differentiate between plants growing in the area but not consumed and those which cannot be consumed because they are absent. To quantify this factor, a regional selection index (RSI) was created to assess differences in edible species selection or rejection among regions. It was obtained by dividing the number of species consumed at a site by the number of species growing there. For instance, the RSI for Sanabria is 0.37 (29/78), since 29 out of 78 available species are used. A regional index for each edible category can be further calculated to assess regional differences in selection among categories. For instance, the RSI for vegetables in Sanabria is 0.19 (5 out of 27 available) versus 0.4 (10/25) for fruits.

## Results and discussion

Additional File 1 illustrates the plant part used, consumption procedure, food use-category and number of informants mentioning each use for the 97 wild edible species reported in the six areas.

All species gathered were authocthonous except *Mespilus germanica *and *Prunus cerasus*, which are now feral. Many species, such as *Corylus avellana*, *Borago officinalis*, *Laurus nobilis*, *Castanea sativa*, *Rubus idaeus*, *Taxus baccata*, *Ulmus minor*, *Mespilus germanica, Prunus avium, Prunus insititia, Ribes uva-crispa *and *Origanum vulgare*, can either be collected in the wild or cultivated in gardens.

Many of the reported uses exist only in the collective memory of the elderly. Most wild fruits, bulbs or flowers mentioned were consumed by children or shepherds as snacks or for amusement on the way to school, or when tending livestock. Some people still pick them on walks to relive the flavours of their childhood.

Food is a very conservative aspect of culture but the erosion on the use and knowledge about wild food plants is higher than that of allotment food plants. The decline in wild food gathering appears to be due to negative connotations, i.e., association with times of scarcity, especially during and after the Civil War (1936–1939). Interestingly, a saying in Piloña – "esi comió berros" (S/he ate *Rorippa nasturtium-aquaticum*) – refers to the starving. Piloña is the only one of the six regions where the above species, one of the most important wild vegetables in Spain and elsewhere [50], is not consumed.

By contrast, wild berries and herbs are still used to make homemade jams (e.g. *Sambucus nigra, Rubus ulmifolius *and *Vaccinium myrtillus)*, desserts and spirits (e.g. *Prunus spinosa, Sideritis hyssopifolia) *for sale as quality local produce.

### Species' Cultural Importance

**Figure 2:**
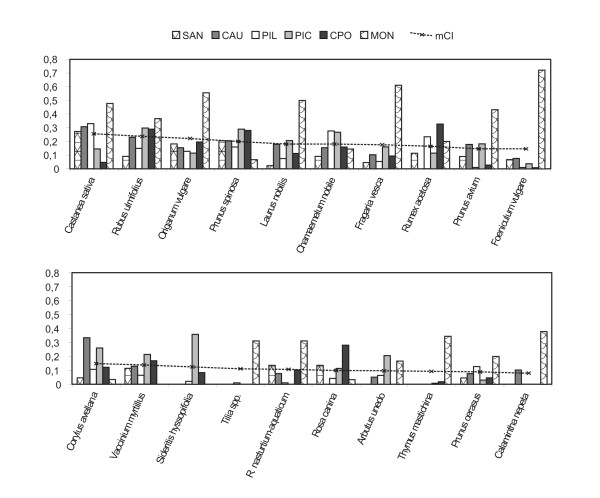
Cultural importance index (CI) of the 20 most relevant species in the northwest of the Iberian Peninsula in descending order by mean value (mCI).

Figure 2 lists, in order of importance, the twenty most culturally important species in the Peninsular northwest according to the MCI, and their CI value in each survey area. The chestnut is the first, second and fifth most important species in Piloña and Sanabria, Caurel and Montesinho, respectively, but is far less significant in Picos, and especially Campoo, where it does not grow spontaneously, being collected the chestnuts from neighbouring areas.

The ten most significant species include fruits (*Castanea sativa*, *Rubus ulmifolius*, *Fragaria vesca, Prunus avium*), seasonings (*Origanum vulgare*, *Laurus nobilis, Foeniculum vulgare*), herbal teas (*Chamaemelum nobile*), liqueurs (*Prunus spinosa*) and vegetables (*Rumex acetosa*). For the six sites as a whole, the species used as fruits are very important, with 4 species in the top 10. Two species used for condiments and one for herbal tea also rank highly. Vegetables are clearly much less important, *Rumex acetosa *ranking eighth and *Rorippa nasturtium-aquaticum *fifteenth.

The least culturally important were those used for their subterranean organs and flowers, the first in each category being *Conopodium *sp. pl. and *Cytinus hypocistis *(40th and 44th in the ranking, respectively).

Table 2 shows the number and percentages of species and of UR among each food-category at each survey site, which indicate that fruits are clearly the most important category in all areas except Montesinho, where it is plants used for seasoning followed by vegetables.

**Table 2:** Number and percentage of wild food species and of use reports (UR) among food- categories at the survey sites

Number of species (N_sp_)														
Food-Category	SAN		CAU		PIL		PIC		CPO		MON		Total	

Vegetables	5	17%	2	10%	7	21%	14	25%	16	28%	16	31%	31	32%
Fruits	10	33%	7	33%	13	38%	28	51%	22	39%	9	18%	32	33%
Seasonings	3	10%	3	14%	2	6%	3	5%	4	7%	14	27%	14	14%
Herbal teas	4	13%	3	14%	6	18%	6	11%	8	14%	13	25%	16	16%
Liqueurs	7	23%	4	19%	6	18%	12	22%	8	14%	11	22%	22	23%
Flowers	1	3%	1	5%	2	6%	2	4%	5	9%	2	4%	8	8%
Subterranean organs	2	7%	1	5%	2	6%	4	7%	4	7%	1	2%	5	5%
Total	30^a^	107%	21^a^	100%	34^a^	112%	55^a^	125%	57^a^	118%	51^a^	129%	97^a^	132%
														

Number of use reports (N_UR_)														

Food-Category	SAN		CAU		PIL		PIC		CPO		MON		Total	

Vegetables	24	20%	6	5%	46	21%	44	8%	103	22%	205	24%	428	18%
Fruits	43	36%	51	45%	84	39%	313	55%	221	47%	167	20%	861	37%
Seasonings	11	9%	17	15%	17	8%	40	7%	37	8%	270	32%	392	17%
Herbal teas	11	9%	15	13%	33	15%	96	17%	40	8%	108	13%	303	13%
Liqueurs	23	19%	19	17%	27	12%	60	11%	32	7%	89	10%	250	11%
Flowers	5	4%	2	2%	5	2%	6	1%	23	5%	10	1%	51	2%
Subterranean organs	4	3%	4	4%	6	3%	11	2%	17	4%	5	1%	47	2%
Total	121	100%	114	100%	218	100%	570	100%	473	100%	854	100%	2332	100%

SAN: Sanabria, Spain; CAU: Caurel, Spain; PIL: Piloña, Spain; PIC: Picos de Europa, Spain; CPO: Campoo, Spain; MON: Montesinho, Portugal.

^a ^A species may be listed in several categories, so these figures may be not the sum of the column. The higher the difference in total percentage over 100%, the more species used in several food categories.

### Differences in CI values for species among the different areas

Figure 2 also indicates appreciable differences among the CI values obtained in the different surveys. Among the ten species with the highest mCI, only *Rumex acetosa *was not cited in all six ethnobotanical surveys. Moreover, most are important in every region. A common cultural background may explain these similarities.

The next ten species include some used only at two or three study sites, e.g. *Calamintha nepeta*, *Tilia *spp., *Sideritis hyssopifolia *and *Thymus mastichina*.

Some species grow in most areas, but only have a high CI value in one of them. For example, the tenth species in the ranking of mCI (see Figure 2), *Foeniculum vulgare*, is the most important species in Montesinho although has a much lower CI in the other areas. *Pterospartum tridentatum*, the tenth species in the CI ranking in Montesinho, is also used only in Piloña. Finally, the vine *Bryonia dioica *is consumed only in Montesinho (Portugal), where it occupies seventh position in the CI ranking despite being quite common at all the survey sites. Also in Montesinho, a larger number of plants used as vegetables and for seasoning occur among those with a high CI.

As mentioned in Methods, mean value was calculated considering only the areas where the species grows since a null value may be due to species not growing there or growing but not being consumed. This mean value therefore takes into consideration species selection or rejection and availability; hence, it is lower for species that grow in the area but are rejected or not considered edible. For instance, as the mCI for *Vaccinium myrtillus*, whose use was mentioned in all five areas where it grows, is obtained by dividing by five, its mCI does not diminish as a result of not growing in one of the areas. Subsequently, the mCIs for *Rorippa nasturtium-aquaticum *or *Crataegus monogyna*, which grow in all six areas but are only consumed in five, were obtained by dividing by 6. The fact that they are not consumed despite being available reduces their mCIs.

Interestingly, Figure 2 also indicates that CI values for edible species in Montesinho are generally higher than for species in the other areas. We can hypothesize that local knowledge of wild edible plants and plant gathering are more widespread in that remote Portuguese region. To analyse this supposition in detail, we can calculate the mean of the CI values for all the species in each area (mCIa) as a measure of botanical knowledge. The mCIa value for Montesinho (0.14) was more than double that of the following values: 0.06 for Campoo, 0.05 for Picos, 0.04 for Caurel, 0.03 for Sanabria and Piloña. This is an exceptional example of great differences in knowledge of wild edible plants among different human groups. Although Sanabria and Montesinho are neighbouring territories sharing a similar environment, the difference is significant, and might be partly explained by a greater loss of knowledge in the former. Moreover, Montesinho has been dependent on natural resources for decades as until the 1990s it was isolated due to the very poor national road network. However, some activities, such as smuggling, periodic migratory farm labouring and selling agricultural produce, have maintained and promoted knowledge of plants.

### Comparison with other Spanish regions

Nearly all the species with a high CI value are also widely consumed throughout Spain and the Mediterranean area. One exception is *Rumex acetosa*, which although the most cited vegetable in Piloña and Campoo, is not as commonly gathered in the rest of Spain [37]. On the other hand, some edible species commonly consumed throughout the Iberian Peninsula, such as *Silene vulgaris *or *Taraxacum officinale*, are seldom collected in some of the study areas despite occurring in all of them.

Some food species eaten in the study areas have scarcely been documented as food plants in the ethnobotanical literature, especially plants whose roots or flowers (e.g., *Crocus nudiflorus*, *Pedicularis schizocalyx, Fritillaria pyrenaica *and *Lamium purpureum*) were used as sweets and *Halimium lasianthum*, whose flowering buds or immature fruits were chewed as a snack. Despite the importance of edible flowers [e.g. [51]], they are often overlooked by researchers, being rejected as merely children's food.

Unusual species occur among plants used for seasoning. In Montesinho, *Physospermum cornubiense *is used for liqueurs and to flavour sweet foods, and leaves and flowers of *Salvia sclarea *for seasoning soups. Also, the flowers and young buds of *Pterospartum tridentatum *are still used to make a local liquid rice dish known as "arroz de carqueja".

### Cultural importance of the families

Regarding the diversity of species gathered, Rosaceae was the most important family, with 17 species. Consumption mainly involves eating ripe berries or making liqueurs (see Additional File 1). Other important families are Lamiaceae, with 13 species, used as condiments and digestive infusions and Asteraceae, with six species being consumed as green vegetables or in infusions. Five species of Polygonaceae were mainly consumed as vegetables and five species of Apiaceae occurred in many use categories. If we compare these figures with those for Spain as a whole [37], the most diverse families of gathered food plants are Asteraceae (92 species), followed by Lamiaceae (53), Rosaceae (34), Apiaceae (25) and Fabaceae (22). These differences are explained by the great importance of wild fruits in the Northwest, most being members of the Rosaceae. In fact, this family is the most diverse in Spain in terms solely of wild fruits. Although almost one-third of the vegetables in Spain belong to the Asteraceae, this category and this family are not so important in the area. The daisy family is also the most diverse family in certain regions of Italy [22,25,52] and the second most diverse in two Turkish areas [13,53].

**Figure 3:**
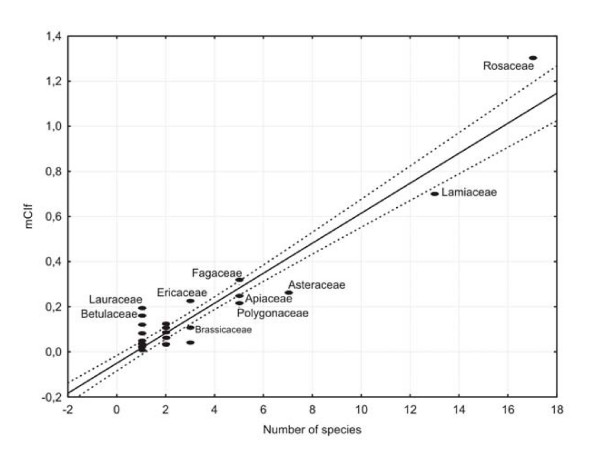
Regression of the cultural importance of the families (mCIf) on the number of species in the family. Discontinuous lines mark the 95% confidence interval.

As we explained in Material and Methods, adding the CI of the species of each family is a good way to measure the cultural importance of the families (CIf). Table 3 shows the most important families in descending order of mCIf. Although a family's cultural importance correlates highly (r = 0.95) with the number of species in each family (see Figure 3) a regression analysis is needed to confirm statistically which families have higher values than expected for the number of species [48].

**Table 3:** Cultural importance of some of the most important families (CIf) in each of the surveyed areas, in descending order of the mean estimated for the whole northwest region (mCIf)

Family	**SAN**	**CAU**	**PIL**	**PIC**	**CPO**	**MON**	mCIf
Rosaceae	0.84	0.80	0.64	1.79	1.73	1.94	1.29
Lamiaceae	0.34	0.31	0.16	0.57	0.39	2.36	0.69
Fagaceae	0.27	0.41	0.36	0.23	0.10	0.48	0.31
Asteraceae	0.09	0.15	0.29	0.33	0.38	0.26	0.25
Apiaceae	0.11	0.18	0.04	0.05	0.15	0.87	0.23
Ericaceae	0.11	0.18	0.13	0.47	0.22	0.17	0.21
Polygonaceae	0.11	0.08	0.23	0.15	0.33	0.36	0.21
Lauraceae	0.02	0.18	0.07	0.21	0.11	0.50	0.18
Betulaceae	0.05	0.33	0.11	0.26	0.12	0.03	0.15
Brassicaceae	0.14	0.08	0.01	0.00	0.10	0.33	0.11

Figure 3 indicates that the plant families with more than 5 species and greater cultural importance as wild food in the northwest of Iberian Peninsula are Rosaceae, Lamiaceae, Fagaceae, Asteraceae, Apiaceae and Polygonaceae. However, only the Rosaceae attains a significantly higher figure for cultural importance (P < 0.05) than that expected for the number of species. This fact remarks once more the high significance of wild fruits category in most of the survey areas, with a large proportion of UR (see Table 2). On the contrary, Lamiaceae, Asteraceae, Apiaceae and Polygonaceae present mCIf values significantly lower than expected.

Finally, a regional particularity that is worthy to comment it is that Lamiaceae is the most important family in Montesinho, according to its CIf (2.35, see Table 3). This is due to the higher relevance of condiments in this area, both in relative number of species and UR, as shown in Table 2.

### Species selection and availability

The relation between species availability and edible use provides interesting insights into food selection criteria in the six areas. As stated in Methods, we established an index called SI to analyse the relationship between species availability and edible use. Table 4 shows the regional selection index for each food-category and the total value for each region. Significant differences appear in the total values of the RSI. In Caurel and Sanabria under 40% of available species were consumed, whereas in Campoo and Montesinho the figure was over 70%.

**Table 4:** Regional Selection Index for each food-category

**Food category**	**SAN**	**CAU**	**PIL**	**PIC**	**CPO**	**MON**	**Mean**
Fruits	0.40	0.29	0.50	0.93	0.79	0.50	0.57
Subterranean organs	0.40	0.33	0.50	0.80	1.00	0.33	0.56
Flowers	0.14	0.20	0.29	0.33	0.71	0.40	0.35
Herbal teas	0.30	0.18	0.43	0.46	0.57	0.86	0.47
Liqueurs	0.44	0.21	0.29	0.63	0.38	0.79	0.47
Vegetables	0.19	0.10	0.27	0.58	0.73	0.64	0.42
Seasoning	0.23	0.30	0.20	0.36	0.33	1.00	0.40

Total RSI	0.37	0.31	0.43	0.68	0.73	0.70	

There are many possible explanations for such differences. Caurel, for example, is a very small isolated region. Although remote areas are commonly thought to yield a greater traditional ecological knowledge, isolation is also associated with lack of information sharing with other regions. Similar conclusions were reached by Milliken and Albert [54] who hypothesised that a high degree of human dispersion as a result of semi-nomadic migration could be responsible for vast knowledge of medicinal plants. Piloña also shows a high percentage of rejection (RSI = 0.43). Its mild climate due to proximity to the sea means that cultivating vegetables and fruit in allotments is more productive, and, consequently, fewer wild plants are needed [40].

On the contrary, the high RSI for Montesinho, Campoo and Picos indicate that a remarkable knowledge of wild edible plants is still employed or at least harboured there. The explanation may lie in cultural reasons such as appreciation of seasoning, vegetables or herbal teas. In the case of Picos, poor communications with other areas, but strong links among local communities, have forged a marked identity, which is also evident in the shared plant uses. The lack of economic development and the taste for certain flavours may explain local appreciation of wild edible plants. Finally, the richness of Campoo could be due to its being a transition area. Its rich flora includes Mediterranean and Atlantic taxa, the easiest route from the plateau of Castile to Santander being across that region. It has therefore received influences from other Cantabrian and Castilian peoples.

The RSI for each food-category further helps to understand the observed differences. If the mean of the RSI for each of the categories is obtained, it is clear that species used for their flowers are much less likely to be selected as edible than fruits or liqueurs (Table 4). That point can be interpreted as cultural pressure or interest to consume wild fruits or liqueurs and as a lack of interest in edible flowers. As mentioned above, the latter plant use is often overlooked by researchers, who do not regard it as proper food, although relevant species may have played a role in human nutrition, especially children's diets.

Wild fruits are widely appreciated in the survey sites, with only Caurel having a RSI below 0.4. The percentage of UR over 40% in Caurel and Campoo and especially in Picos (55%), shown in Table 2, corroborate this fact. Low productivity of cultivated fruit trees, lack of money, bad communications, and limited fruit supply in the markets, especially in winter, meant people could not buy commercial fruits. They depended on countryside fruits such as *Mespilus germanica*, *Sorbus aria, Malus sylvestris *or *Prunus spinosa*. Although not very productive, these plants are well adapted to local weather conditions. Significantly, only a few species of wild fruits are rejected in four or five areas; they include *Amelanchier ovalis*, rare in the study areas, and the bitter and unpleasant acorns of *Quercus robur *and *Quercus petraea*. Although once an essential part of the diet [55-57], the latter two are now regarded as animal food.

Unsurprisingly, a number of wild vegetables appear among the unselected species since in most of the six survey areas diets are rich in beans, cabbage and potato, but quite poor in vegetables, with only a few condiments being used [38,40,41].

Montesinho, however, presents remarkable features, with wild condiments (32% of UR) and vegetables (24% of UR) playing a very important role (see Table 2). Condiments such as *Foeniculum vulgare, Pterospartum tridentatum, Calamintha nepeta, Lavandula stoechas *or *Thymus mastichina *were used for apparent variation, mainly in soups and purees. Wild vegetables were used to garnish meat or fish dishes. Sometimes, during periods of scarcity, edible greens were eaten with potatoes as a substitute for meat and fish. Campoo also presents a higher percentage of vegetable selection, probably due to its closer relationship with Central Spain, where more vegetables are consumed [37].

## Conclusion

Our comparison indicates that patterns of wild edible plant usage appear to depend mainly on socio-cultural factors rather than biological ones such as climate or richness of the wild edible flora. Availability of running water, free time to tend allotments, better communications and information exchange, direct contact with nature in everyday life, cultural values, fads and taste preferences are some of the factors that explain why wild plants are either consumed or rejected.

There is a clear preference for wild edible fruits that are consumed raw or used to make jams and liqueurs. By contrast, people in most of the study areas reject many available wild vegetables.

Some wild species are still gathered, including plants historically consumed in all areas with a high number of URs. They are the most important species in each use-category (fruits, vegetables, infusions or liqueurs), grow in all the survey sites and if not easily available from the wild, they are often cultivated. These "key plants" represent the core wild food flora.

Many wild edible plants are regarded as famine food and are no longer gathered. In rural Spain and Portugal they are often considered to be old fashioned, unprofitable, or too time-consuming, cultivated plants or bought food being consumed in preference.

Radical changes in the way of life of rural people in Spain and Portugal have severely eroded knowledge and customs relating to the exploitation and management of most wild resources. This rich element of biocultural diversity needs, therefore, to be studied before it is too late [58].

New trends relating to these resources can be traced in other Mediterranean countries, and the social significance and meaning of some are being reinterpreted. Although in general terms wild edible plants often have a stigma attached to them, being regarded as poor people's food, some are increasingly popular as delicacies, local specialities, gourmet food and local food that reflects regional identity. For example, both demand and supply are increasing in the cases of *Asparagus acutifolius *in many European regions, *Muscari comosum *in Italy and *Sideritis hyssopifolia *in Cantabria [20,37,59,60]

Movements such as Slow Food [61] and chefs' interest in offering new flavours and dishes can play a crucial role in boosting the social importance of such resources. Moreover, the way local people perceive and use their resources plays an important role in their conservation. Changes can lead to unpredictable consequences to their sustainable development.

## Competing interests

The author(s) declare that they have no competing interests.

## Authors' contributions

MP, EB, ES, AC and JL designed and carried out the field work; MP and JT wrote the paper, which was revised and improved by the comments of all the authors. RM coordinated, helped in the design of most of the surveys and supervised the paper. All authors read and approved the final manuscript.

## Supplementary Material

Additional File 1 Wild food plants traditionally consumed and number of informants that mention them. Table describing the wild food plants consumed in the region, including the number of informants that mentioned them. Click here for file

## References

[B1] Nebel S, Pieroni A, Heinrich M (2006). Ta chòrta: Wild edible greens used in the Graecanic area in Calabria, southern Italy. Appetite.

[B2] Heinrich M, Müller WE, Galli C (Eds.) (2006). Local Mediterranean Food Plants and Nutraceuticals.

[B3] Delang CO (2006). Not just minor forest products: The economic rationale for the consumption of wild food plants by subsistence farmers. Ecological Economics.

[B4] Vitalini S, Grande S, Visioli F, Agradi E, Fico G, Tome F (2006). Antioxidant activity of wild plants collected in Valsesia, an alpine region of Northern Italy. Phytotherapy Research.

[B5] Halwart M (2006). Biodiversity and nutrition in rice-based aquatic ecosystems. Journal of Food Composition and Analysis.

[B6] Redzic SJ (2006). Wild edible plants and their traditional use in the human nutrition in Bosnia-Herzegovina. Ecology of Food and Nutrition.

[B7] Johns T, Eyzaguirre PB (2006). Linking biodiversity, diet and health in policy and practice. Proceedings of the Nutrition Society.

[B8] Delang CO (2006). Indigenous systems of forest classification: Understanding land use patterns and the role of NTFPs in shifting cultivators' subsistence economies. Environmental Management.

[B9] Ansari NM, Houlihan L, Hussain B, Pieroni A (2005). Antioxidant activity of five vegetables traditionally consumed by South-Asian migrants in Bradford, Yorkshire, UK. Phytotherapy Research.

[B10] Balemie K, Kebebew F (2006). Ethnobotanical study of wild edible plants in Derashe and Kucha Districts, South Ethiopia. Journal of Ethnobiology and Ethnomedicine.

[B11] Arenas P (2003). Etnografía y Alimentación entre los Toba-Ñachilamole#ek y Wichí-Lhuku´tas del Chaco Central (Argentina).

[B12] El SN, Karakaya S (2004). Radical scavenging and iron-chelating activities of some greens used as traditional dishes in Mediterranean diet. International Journal of Food Sciences and Nutrition.

[B13] Ertug F (2004). Wild edible plants of the Bodrum Area (Mugla,Turkey). Turkish Journal of Botany.

[B14] Shrestha PM, Dhillion SS (2006). Diversity and traditional knowledge concerning wild food species in a locally managed forest in Nepal. Agroforestry Systems.

[B15] Lockett CT, Calvert CC, Grivetti LE (2000). Energy and micronutrient composition of dietary and medicinal wild plants consumed during drought. Study of rural Fulani, Northeastern Nigeria. International Journal of Food Sciences and Nutrition.

[B16] Kuhnlein H, Erasmus B, Creed-Kanashiro H, Englberger L, Okeke C, Turner N, Allen L, Bhattacharjee L (2006). Indigenous peoples' food systems for health: finding interventions that work. Public Health and Nutrition.

[B17] Heinrich M, Leonti M, Nebel S, Peschel W, Pieroni A, Smith F, Rivera D, Obon C, Inocencio C, Verde A, Fajardo J, Llorach R, Muller WE, Eckert GP, Schaffer S, Schmitt-Schillig S, Antonopoulou S, Kypriotakis Z, Manios Y, Nomikos T, Kaliora A, Sidossis L, Galli C, Visioli F, Grande S, Bogani P, de Saizieu A, Fluhmann B, D'Orazio D, Fowler A, Koj A, Bereta J, Dulak J, Guzdek A, Kapiszewska M (2005). Understanding local Mediterranean diets: A multidisciplinary pharmacological and ethnobotanical approach. Pharmacological Research.

[B18] Luczaj L, Szymanski W (2007). Wild vascular plants gathered for consumption in the Polish countryside: a review. Journal of Ethnobiology and Ethnomedicine.

[B19] Pieroni A, Nebel S, Quave C, Münz H, Heinrich M (2002). Ethnopharmacology of liakra: traditional weedy vegetables of the Arbëreshë of the Vulture area in southern Italy. Journal of Ethnopharmacology.

[B20] Pieroni A, Nebel S, Santoro RF, Heinrich M (2005). Food for two seasons: Culinary uses of non-cultivated local vegetables and mushrooms in a south Italian village. International Journal of Food Sciences and Nutrition.

[B21] Bonet MA, Vallès J (2002). Use of non-crop food vascular plants in Montseny biosphere reserve (Catalonia, Iberian Peninsula). International Journal of Food Sciences and Nutrition.

[B22] Guarrera PM, Salerno G, Caneva G (2006). Food, flavouring and feed plant traditions in the Tyrrhenian sector of Basilicata, Italy. Journal of Ethnobiology and Ethnomedicine.

[B23] Della A, Paraskeva-Hadjichambi D, Hadjichambis AC (2006). An ethnobotanical survey of wild edible plants of Paphos and Larnaca countryside of Cyprus. Journal of Ethnobiology and Ethnomedicine.

[B24] Guarrera PM (2003). Food medicine and minor nourishment in the folk traditions of Central Italy (Marche, Abruzzo and Latium). Fitoterapia.

[B25] Pardo-de-Santayana M, Tardío J, Morales R (2005). The gathering and consumption of wild edible plants in Campoo (Cantabria, Spain). International Journal of Food Sciences and Nutrition.

[B26] Tardío J, Pascual H, Morales R (2005). Wild food plants traditionally used in the province of Madrid, central Spain. Economic Botany.

[B27] Ghirardini MP, Carli M, Del Vecchio N, Rovati A, Cova O, Valigi F, Agnetti G, Macconi M, Adamo D, Traina M, Laudini F, Marcheselli I, Caruso N, Gedda T, Donati F, Marzadro A, Russi P, Spaggiari C, Bianco M, Binda R, Barattieri E, Tognacci A, Girardo M, Vaschetti L, Caprino P, Sesti E, Andreozzi G, Coletto E, Belzer G, Pieroni A (2007). The importance of a taste: A comparative study on wild food plants consumption in twenty-one local communities in Italy. Journal of Ethnobiology and Ethnomedicine.

[B28] Lentini F, Venza F (2007). Wild food plants of popular use in Sicily. Journal of Ethnobiology and Ethnomedicine.

[B29] Ladio AH, Lozada M (2003). Comparison of wild edible plant diversity and foraging strategies in two aboriginal communities of northwestern Patagonia. Biodiversity and Conservation.

[B30] Díaz-Betancourt M, Ghermandi L, Ladio AH, López-Moreno IR, Raffaele E, Rapoport. EH (1999). Weeds as a source for human consumption. A comparison between tropical and temperature Latin America. Revista de Biología Tropical.

[B31] Leonti M, Nebel S, Rivera D, Heinrich M (2006). Wild gathered food plants in the European Mediterranean: A comparative analysis. Economic Botany.

[B32] Goodman SM, Hobbs JJ (1988). The ethnobotany of the Egyptian Eastern Desert: a comparison of common plant usage between two culturally distinct Bedouin groups. Journal of Ethnopharmacology.

[B33] Leporatti ML, Ivancheva S (2003). Preliminary comparative analysis of medicinal plants used in the traditional medicine of Bulgaria and Italy. Journal of Ethnopharmacology.

[B34] Moerman DE, Pemberton RW, Kiefer D, Berlin B (1999). A comparative analysis of five medicinal floras. Journal of Ethnobiology.

[B35] Pieroni A, Quave CL (2005). Traditional pharmacopoeias and medicines among Albanians and Italians in southern Italy: A comparison. Journal of Ethnopharmacology.

[B36] Rossato SC, Leitao-Filho HD, Begossi A (1999). Ethnobotany of Caicaras of the Atlantic Forest coast (Brazil). Economic Botany.

[B37] Tardío J, Pardo-de-Santayana M, Morales R (2006). Ethnobotanical review of wild edible plants in Spain. Botanical Journal of the Linnean Society.

[B38] Pardo-de-Santayana M (2003). Las plantas en la cultura tradicional de la Antigua Merindad de Campoo. PhD thesis.

[B39] Lastra JJ (2003). Etnobotánica en el Parque Nacional de Picos de Europa.

[B40] San Miguel E (2004). Etnobotánica de Piloña (Asturias). Cultura y saber popular sobre las plantas en un concejo del Centro-Oriente Asturiano. PhD thesis.

[B41] Blanco E (1996). El Caurel. Las plantas y sus habitantes. Estudio etnobotánico de la Sierra del Caurel (Lugo): la importancia de las plantas para nuestros antepasados.

[B42] Blanco E, Diez J (2005). Guía de Flora de Sanabria, Carballeda y Los Valles. Catálogo de Etnoflora selecta.

[B43] Carvalho AMP (2005). Etnobotánica del Parque Natural de Montesinho, Plantas, tradición y saber popular en un territorio del nordeste de Portugal.. PhD thesis.

[B44] Alexiades MN (1996). Selected Guidelines for Ethnobotanical Research: A Field Manual. Advances in Economic Botany.

[B45] Kufer J, Förther H, Pöll E, Heinrich M (2005). Ethnobotany of the Ch'orti'. Journal of Pharmacy and Pharmacology.

[B46] Tardío J, Pardo-de-Santayana M Cultural importance indices: a comparative analysis based on of the useful wild plants of southern Cantabria (Northern Spain). Economic Botany.

[B47] Reyes-García V, Huanca T, Vadez V, Leonard W, Wilkie D (2006). Cultural, practical, and economic value of wild plants: A quantitative study in the Bolivian Amazon. Economic Botany.

[B48] Phillips O, Gentry AH (1993). The Useful Plants of Tambopata, Peru .1. Statistical Hypotheses Tests with a New Quantitative Technique. Economic Botany.

[B49] Galeano G (2000). Forest use at the Pacific coast of Choco, Colombia: A quantitative approach. Economic Botany.

[B50] Coupland F (1989). Le regal vegetal. Plantes sauvages comestibles. Encyclopedie des plantes comestibles de l'Europe, Vol 1.

[B51] Morton JF, Alvarez E, Quinonez C (1990). Loroco, Fernaldia-Pandurata (Apocynaceae) - a Popular Edible Flower of Central-America. Economic Botany.

[B52] Pieroni A (1999). Gathered wild food plants in the Upper Valley of the Serchio River (Garfagnana), Central Italy. Economic Botany.

[B53] Ertug F (2000). An ethnobotanical study in Central Anatolia (Turkey). Economic Botany.

[B54] Milliken W, Albert B (1997). The use of medicinal plants by the Yanomami indians of Brazil, Part II. Economic Botany.

[B55] Zapata L (2000). La recolección de plantas silvestres en la subsistencia mesolítica y neolítica. Datos arqueobotánicos del País Vasco. Complutum.

[B56] Mason S (1992). Acorns in human subsistence.

[B57] Pereira Sieso J (2002). Bellotas, el alimento de la Edad de Oro. Arqueoweb.

[B58] Vallès J, Bonet MA, Agelet A (2004). Ethnobotany of Sambucus nigra L. in Catalonia (Iberian peninsula): The integral exploitation of a natural resource in mountain regions. Economic Botany.

[B59] Casoria P, Menale B, Muoio R (1999). Muscari comosum, Liliaceae, in the Food Habits of South Italy. Economic Botany.

[B60] Pardo-de-Santayana M, Blanco E, Morales R (2005). Plants known as "té" (tea) in Spain. An ethno-pharmaco-botanical review.. Journal of Ethnopharmacology.

[B61] Slow food. http://www.slowfood.com.

