# Supplementary material for: Traditional knowledge of wild edible plants used in the northwest of the Iberian Peninsula (Spain and Portugal): a comparative study
Source: J Ethnobiol Ethnomed. 2007 Jun 7;3:27. doi: 10.1186/1746-4269-3-27 (PMC1904191; doi:10.1186/1746-4269-3-27)
Supplement: Additional File 1 — Wild food plants traditionally consumed and number of informants that mention them. Table describing the wild food plants consumed in the region, including the number of informants that mentioned them. [file 1746-4269-3-27-S1.pdf]

Additional file 1. Wild food plants traditionally consumed and number of informants that mention each food-use in the survey areas. SAN: Sanabria; CAU: Caurel; PIL: Piloña; PIC: Picos de Europa; CPO: Campoo; MON: Montesinho. Present but not mentioned in the area (●), absent (–).

**Family/Species**	**SAN**	**CAU**	**PIL**	**PIC**	**CPO**	**MON**	**Food use-categor**	**Part used, way of consumption**
APIACEAE								
Apium nodiflorum (L.) Lag.	2	●	3	●	–	●	Vegetables	Tender leaves and stems, raw in salads
Conopodium sp. pl. (*C. majus* (Gouan) Loret, *C. pyrenaeum* (Loisel.) Miégev., *C. subcarneum* (Boiss. & Reut.) Boiss. & Reut.)	●	4	●	1	9	5	Subterranean organs	Tubers, raw as a snack
Foeniculum vulgare Mill.	3	3	1	2	●	10	Liqueurs	Aerial part or seeds for liqueurs
	●	●	●	1	1	●	Vegetables	Tender leaves and stems, raw in salads or stewed
	●	●	●	●	●	32	Seasoning	Seeds for seasoning soups and stews
	●	●	●	2	●	23	Herbal teas	Aerial part or seeds for herbal teas
Physospermum cornubiense (L.) DC.	–	●	●	–	●	3	Seasoning	Fruits, for flavouring different sorts of cakes made with chestnuts
	–	●	●	–	●	2	Liqueurs	Tender stems for liqueurs
	–	●	●	–	●	3	Herbal teas	leaves for herbal teas
Scandix australis L.	–	–	–	–	4	–	Vegetables	Tender leaves and stems, raw as a snack
	–	–	–	–	2	–	Liqueurs	Tender leaves and stems for liqueurs
ASTERACEAE								
Achillea millefolium L.	●	●	1	6	3	2	Herbal teas	Inflorescences, as herbal tea
Chamaemelum nobile (L.) All.	4	6	26	34	17	13	Herbal teas	Inflorescences, as herbal tea
	●	●	●	1	●	●	Liqueurs	Inflorescences for liqueurs
Helichrysum stoechas (L.) Moench	–	–	–	●	5	●	Herbal teas	Inflorescences, as herbal tea
Hypochoeris radicata L.	●	●	●	●	2	●	Vegetables	Basal leaves, raw as a snack
Matricaria discoidea DC.	●	●	●	●	●	8	Herbal teas	Inflorescences, as herbal tea
Taraxacum officinale Weber	●	●	●	2	5	●	Vegetables	Basal leaves, raw as a snack
Tragopogon pratensis L.	●	●	●	●	9	–	Vegetables	Tender leaves and stems, raw as a snack
BERBERIDACEAE								
Berberis vulgaris L.	●	●	●	3	●	–	Vegetables	Tender leaves, raw as a snack
BETULACEAE								
Corylus avellana L.	2	13	10	34	13	3	Fruits	Fruits, eaten raw, dried, added to cakes
BORAGINACEAE								

Borago officinalis L.	●	–	●	1	1	22	Vegetables	Basal leaves, stewed
Lithospermum officinale L.	–	–	●	●	4	●	Herbal teas	Aerial part for herbal teas
BRASSICACEAE								
Rorippa nasturtium-aquaticum (L.) Hayek	6	3	1	●	11	28	Vegetables	Tender leaves and stems; raw in salads or stewed in soups
Sinapis arvensis L.	●	●	●	●	–	2	Seasoning	Seeds milled as a condiment
CANNABACEAE								
Humulus lupulus L.	–	●	●	–	●	9	Liqueurs	Immature female inflorescences for making liqueurs
CAPRIFOLIACEAE								
Sambucus nigra L.	●	●	1	5	6	3	Fruits	Fruits, for making jams or syrups
	●	●	●	1	●	3	Liqueurs	Fruits, for making liqueurs
Viburnum lantana L.	–	–	●	–	13	–	Fruits	Fruits, eaten raw as snack, or stored wrapped in straw
CARYOPHYLLACEAE								
Silene vulgaris (Moench) Garcke	●	●	●	1	1	●	Vegetables	Leaves and young stems, stewed in omelettes and other dishes
CHENOPODIACEAE								
Chenopodium murale L.	●	–	–	–	–	2	Vegetables	Leaves, stewed in soups
CISTACEAE								
Halimium lasianthum subsp. *alyssoides* (Lam.) Greuter	–	–	–	–	–	3	Vegetables	Young floral buds and immature fruits, chewed raw as a snack
CUCURBITACEAE								
Bryonia dioica Jacq.	●	●	●	●	●	37	Vegetables	Young shoots with leaves, stewed
CUPRESSACEAE								
Juniperus communis subsp. *alpina* (Suter) Čelak	4	●	●	●	●	–	Liqueurs	Fruits, for making liqueurs
ERICACEAE								
Arbutus unedo L.	●	2	4	26	–	●	Fruits	Fruits, eaten raw
	●	●	2	1	–	15	Liqueurs	Fruits for making liqueurs
Arctostaphylos uva-ursi (L.) Spreng.	–	–	–	7	5	–	Fruits	Fruits, eaten raw as snack
Vaccinium myrtillus L.	4	●	6	22	18	–	Fruits	Fruits, eaten raw and for making jam
	1	5	●	6	●	–	Liqueurs	Fruits, for making liqueurs
FABACEAE								
Pterospartum tridentatum (L.) Willk.	●	●	●	●	●	21	Seasoning	Inflorescences, as a condiment for chicken and rabbit meat
	●	●	●	●	●	6	Vegetables	Young shoots, stewed with rice
	●	●	1	●	●	10	Herbal teas	Flowered aerial part as herbal tea
Trifolium alpinum L.	1	–	–	8	3	–	Subterranean organs	Roots, chewed and sucked
Trifolium pratense L.	●	●	●	2	1	●	Flowers	Flowers sucked
FAGACEAE								

Castanea sativa Mill.	12	12	31	19	5	43	Fruits	Fruits, eaten raw, dried, roasted or added as a condiment for stews, and desserts, sometimes ground into flour
Fagus sylvatica L.	–	4	3	4	6	–	Fruits	Fruits, eaten raw or for extracting oil
Quercus ilex L. [subsp. ballota (Desf.) Samp.]	●	●	●	2	●	●	Fruits	Fruits, roasted
Quercus petraea (Matt.) Liebl.	●	●	●	3	●	–	Fruits	Fruits, roasted
Quercus robur L.	●	●	●	2	–	●	Fruits	Fruits, roasted
GROSSULARIACEAE								
Ribes alpinum L.	–	–	●	7	14	–	Fruits	Fruits, eaten raw
Ribes uva-crispa L.	–	●	●	1	11	–	Fruits	Fruits, eaten raw
IRIDACEAE								
Crocus nudiflorus Sm.	●	–	3	●	–	–	Subterranean organs	Bulbs, raw as a snack
Romulea bulbocodium (L.) Sebast. & Mauri	●	●	●	1	4	●	Subterranean organs	Bulbs, raw as a snack
LAMIACEAE								
Calamintha nepeta (L.) *Savi*	●	4	●	●	●	17	Seasoning	Aerial part, as a condiments for soups and stews
	●	●	●	●	●	17	Herbal teas	Aerial part, as herbal tea
Lamium maculatum L.	●	●	1	4	3	●	Flowers	Flowers sucked
Lamium purpureum L.	●	●	●	●	●	7	Flowers	Flowers sucked
Lavandula stoechas L.	●	–	–	●	●	21	Seasoning	Aerial part for seasoning rabbit meat
Mentha aquatica L.	●	–	●	–	●	6	Herbal teas	Aerial part, as herbal tea
Mentha pulegium L.	3	●	●	●	1	6	Herbal teas	Inflorescences, as herbal teas
	●	●	●	●	●	4	Liqueurs	Inflorescences, for makings liqueurs
	●	●	●	●	●	23	Seasoning	Aerial part, as a condiment for soups
Mentha suaveolens Ehrh.	●	●	●	●	●	9	Seasoning	Aerial part, as a condiment for soups
Origanum vulgare L.	8	6	10	12	20	48	Seasoning	Inflorescences, for seasoning pork (black pudding, "chorizo", marinated pork), stews and roasted meat
	●	●	2	3	1	2	Herbal teas	Inflorescences, as herbal tea
Salvia sclarea L.	●	–	–	–	–	4	Seasoning	Leaves and inflorescences, for seasoning soups
Sideritis hyssopifolia L.	–	●	2	45	9	–	Herbal teas	Inflorescences, as herbal tea
	–	●	●	2	●	–	Liqueurs	Inflorescences for making liqueurs
Thymus mastichina (L.) L.	●	–	–	1	2	31	Seasoning	Inflorescences, for seasoning
Thymus pulegioides L.	2	2	●	6	3	3	Herbal teas	Inflorescences, as herbal tea
	●	●	●	1	●	2	Seasoning	Inflorescences, for seasoning fish and meat stews and "chorizo"

Thymus zygis Loefl. ex L.	2	–	–	–	3	12	Seasoning	Inflorescences, for seasoning stews and meat
LAURACEAE								
Laurus nobilis L.	1	7	7	27	12	45	Seasoning	Leaves, for seasoning stews and "chouriço"
LILIACEAE								
Allium spp.	●	●	●	1	1	●	Vegetables	Bulbs, for seasoning stews
Fritillaria pyrenaica L.	●	–	●	●	5	–	Flowers	Flowers sucked
Merendera montana (L.) Lange	3	●	3	1	1	●	Subterranean organs	Tubers, raw as a snack
MALVACEAE								
Malva sylvestris L.	●	●	●	2	2	5	Vegetables	immature fruits, raw as a snack
Malva tournefortiana L.	●	–	–	–	–	3	Vegetables	immature fruits, raw as a snack
NYMPHAEACEAE								
Nymphaea alba L.	3	–	–	–	–	–	Fruits	Fruits, eaten raw
OXALIDACEAE								
Oxalis acetosella L.	●	–	7	1	1	–	Vegetables	Leaves, raw as a snack
POLYGONACEAE								
Rumex acetosa L.	5	●	22	15	35	18	Vegetables	Basal leaves, raw as a snack, in salads or stewed in soups
Rumex acetosella L.	●	3	●	●	●	10	Vegetables	Leaves, raw as a snack and in salads
Rumex induratus Boiss. & Reut.	●	–	–	–	–	3	Vegetables	Leaves, raw in salads
Rumex obtusifolius L.	●	●	●	1	●	1	Vegetables	Leaves, stewed
Rumex scutatus L.	–	–	●	3	–	–	Vegetables	Leaves, raw as a snack
PORTULACACEAE								
Montia fontana L.	4	●	●	●	–	22	Vegetables	Tender leaves and stems, raw in salads
Portulaca oleracea L.	●	–	●	●	–	20	Vegetables	Tender leaves and stems, stewed
PRIMULACEAE								
Primula acaulis (L.) L.	●	2	4	●	●	●	Flowers	Flowers sucked
Primula elatior (L.) L.	–	–	–	●	3	–	Flowers	Flowers sucked
RAFFLESIACEAE								
Cytinus hypocistis (L.) L.	5	●	●	–	–	3	Flowers	Flowers sucked
ROSACEAE								
Amelanchier ovalis Medik.	●	–	●	●	2	●	Fruits	Fruits, eaten raw
Crataegus monogyna Jacq.	4	●	1	20	17	3	Fruits	Fruits, eaten raw
Fragaria vesca L.	2	4	5	21	10	30	Fruits	Fruits, eaten raw and for making jam or desserts
	●	●	●	●	●	25	Liqueurs	Fruits, for making liqueurs
Malus sylvestris (L.) Mill.	●	●	●	4	16	●	Fruits	Fruits, eaten raw as a snack or stored and used as dessert
	2	●	●	14	4	●	Liqueurs	Fruits, for making liqueurs or cider
Mespilus germanica L.	–	–	●	4	5	–	Fruits	Fruits, eaten raw after stored
	–	–	1	●	●	–	Liqueurs	Fruits, for making liqueurs
Prunus avium L.	4	7	1	21	3	37	Fruits	Fruits, eaten raw as dessert and for making jam
	●	●	●	3	●	2	Liqueurs	Fruits, for making liqueurs

Prunus cerasus L.	●	●	●	●	●	11	Fruits	Fruits, for making jams
	2	3	12	4	5	7	Liqueurs	Fruits, for making liqueurs
Prunus insititia L	–	–	●	2	7	–	Fruits	Fruits, eaten raw
	–	–	●	●	4	–	Liqueurs	Fruits, for making liqueurs
Prunus spinosa L.	●	●	6	17	16	●	Fruits	Fruits, eaten raw after stored
	9	8	9	21	14	6	Liqueurs	Fruits, for making liqueurs
Pyrus cordata Desv.	●	●	4	7	5	●	Fruits	Fruits, eaten raw after stored
	●	●	●	●	1	●	Liqueurs	Fruits, for making liqueurs
Rosa canina L	●	●	4	4	11	●	Vegetables	Young shoots, raw as a snack
	6	●	●	10	18	3	Fruits	Fruits, eaten raw
	●	●	●	1	1	●	Liqueurs	Fruits, for making liqueurs
Rubus idaeus L.	2	●	●	7	1	9	Fruits	Fruits, eaten raw as dessert
	2	●	●	●	●	9	Liqueurs	Fruits, for making liqueurs
Rubus ulmifolius Schott	●	●	5	6	14	●	Vegetables	Young shoots, raw as a snack
	4	9	7	29	16	27	Fruits	Fruits, eaten raw as dessert or for making jams
	●	●	2	4	1	6	Liqueurs	Fruits, for making liqueurs or other beverages
Sorbus aria (L.) Crantz	●	●	3	27	14	–	Fruits	Fruits, eaten raw (sometimes as dessert)
Sorbus aucuparia L.	●	●	–	2	●	●	Fruits	Fruits, eaten raw
Sorbus intermedia (Ehrh.) Pers.	●	–	–	3	–	–	Fruits	Fruits, eaten raw
Sorbus torminalis (L.) Crantz	●	●	●	4	●	●	Fruits	Fruits, eaten raw
SCROPHULARIACEAE								
Pedicularis schizocalyx (Lange) Steininger	●	–	●	–	11	–	Flowers	Flowers sucked
TAXACEAE								
Taxus baccata L.	●	●	3	3	●	●	Fruits	The aril of the seed, eaten raw
TILIACEAE								
Tilia platyphyllos Scop., *T. cordata* Mill.	–	–	1	●	–	28	Herbal teas	Flowers, as herbal tea
ULMACEAE								
Ulmus minor Mill.	7	●	●	●	●	●	Vegetables	immature fruits, raw as a snack
URTICACEAE								
Urtica dioica L.	●	●	4	3	3	21	Vegetables	Tender leaves and stems, stewed
Urtica membranacea Poir.	–	–	●	–	–	12	Vegetables	Tender leaves and stems, stewed
VALERIANACEAE								
Valerianella carinata Loisel.	●	–	●	–	2	–	Vegetables	Basal leaves, raw in salads
